# Prevalence of Paediatric Surgical Conditions in Eastern Uganda: A Cross-Sectional Study

**DOI:** 10.1007/s00268-021-06378-9

**Published:** 2022-01-01

**Authors:** Mary Margaret Ajiko, Viking Weidman, Pär Nordin, Andreas Wladis, Jenny Löfgren

**Affiliations:** 1 Department of Molecular Medicine and Surgery, Karolinska Institute, Solna, Sweden; 2 Soroti Regional Referral Hospital, Box 289, Soroti, Uganda; 3 Uppsala University, Uppsala, Sweden; 4 Department of Surgery and Perioperative Sciences, University of Umeå, Umeå, Sweden; 5 Department of Biomedical and Clinical Sciences, Linköping University, Linköping, Sweden

## Abstract

**Background:**

The role of surgery in global health has gained greater attention in recent years. Approximately 1.8 billion children below 15 years live in low- and middle-income countries (LMIC). Many surgical conditions affect children. Therefore, paediatric surgery requires specific emphasis. Left unattended, the consequences can be dire. Despite this, there is a paucity of data regarding prevalence of surgical conditions in children in LMIC. The present objective was to investigate the prevalence of paediatric surgical conditions in children in a defined geographical area in Eastern Uganda.

**Method:**

A cross-sectional study was carried out in the Iganga-Mayuge Health and Demographic Surveillance Site located in Eastern Uganda. Through a two-stage, cluster-based sampling process, 490 households from 49 villages were randomly selected, generating a study population of 1581 children. The children’s caregivers were interviewed, and the children were physically examined by two medical doctors to identify any surgical conditions.

**Results:**

The interview was performed with 1581 children, and 1054 were physically examined. Among these, the overall prevalence of any surgical condition was 16.0 per cent (*n* = 169). Of these, 39 per cent had an unmet surgical need (66 of 169). This is equivalent to a 6.3 per cent prevalence of current unmet surgical need. The most common groups of surgical condition were congenital anomalies and trauma-related conditions.

**Conclusion:**

Surgical conditions in children are common in eastern Uganda. The unmet need for surgery is high. With a growing population, the need for paediatric surgical capacity will increase even further. The health care system must be reinforced to provide services for children with surgical conditions if United Nations Sustainability Development Goal 3 is to be achieved by 2030.

**Supplementary Information:**

The online version contains supplementary material available at 10.1007/s00268-021-06378-9.

## Introduction

In 2015, the UN Sustainability Development Goals (SDG) were defined and represent a continuation of the Millennium Development Goals. Child health remains at the core of the SDG3 “to ensure healthy lives for all at all ages”. Specifically, preventable deaths among newborns and in children below 5 years should end globally by 2030 [1]. To reach this ambitious goal, preventive measures along with universal health coverage are needed. Surgical services are an essential part of universal health care coverage, but surgical care for children is an area of child health that is often overlooked.

Interview-based research using the Surgeons’ Overseas Assessment of Surgical Need (SOSAS) methodology and tool has been carried out to define the burden of paediatric surgical conditions. In Rwanda, Sierra Leone, Nepal and Uganda, 19% of children overall had a surgical need and 62% of these had at least one unmet need [2]. In Uganda, the prevalence of untreated surgical conditions was 7.4% in 2016, with trauma, wounds, acquired deformities and burns the most common groups of conditions [3].

These studies did not include physical examination of the children, and the diagnoses were not confirmed. It is not possible to conclude how far self-reported health issues are consistent with clinical findings. To better define the burden of surgical disease, including the level of disability that they cause, population-based research including physical examination for accurate diagnosis is needed. The present aim was to define the burden of surgical conditions, the prevalence of unmet surgical need and the level of disability caused by these conditions in children in a defined population in eastern Uganda.

## Methods

### Study design

This was a cross-sectional, cluster-based study.

### Study setting

Uganda is a low-income country in sub-Saharan Africa with a population of 41.6 million of whom almost 50% were under 15 years old in 2019 [4, 5]. This cross-sectional study was carried out at the Health and Demographic Surveillance site in Iganga and Mayuge Districts (I/M HDSS) in Eastern Uganda between August and October 2019. The I/M HDSS is a geographically defined area where births, deaths, cause of deaths and population migration have been regularly monitored since 2005 at this surveillance site. The I/M HDSS covers 65 villages and 18,004 households. In 2018, the population was 94,568 people, of whom 54.6% (51,634) were below 18 years [6].

### Study population

Children (below 18 years) resident in the I/M HDSS at the time of the study were eligible study participants. A person is categorized as a resident of the area after having lived there for at least 4 months. Newborns under the age of 4 months residing in a household in the HDSS are also considered residents.

### Definitions

Disability is any restriction or lack (resulting from an impairment) of ability to perform an activity in the manner or within the range considered normal for a human being [7, 8]. Disability was graded from 1 to 7 (Appendix 1 in ESM). The unmet need for surgery was defined as the proportion of children with surgical conditions that have not received adequate necessary surgical treatment. The treatment recommendation for asymptomatic umbilical hernia was watchful observation until 5 years of age [9]. Thus, a child below five with an asymptomatic umbilical hernia was defined as having a surgical condition but currently not needing surgery.

### Sample size and sample selection

The I/M HDSS has information about the villages and the households in a database updated through routine surveillance data collection performed twice a year. The information includes the head of each household and the name, age and sex of children residing in the household. The sample selection was done through a two-stage cluster process by a statistician working for the I/M HDSS. In the first stage, 49 of the 65 villages were randomly selected. Thereafter, ten households based on structure number (each building in the I/M HDSS has a unique number), within each village were randomly selected. This generated a total of 490 households. All children in the sample households whose names were in the database were included in the study as well as those born after the enumeration.

The number of villages (clusters) to include was calculated according to the B. S. Wood et al. formula below [10].

$$C = \frac{p(1 - p)D}{{s^{2} b}}\quad {\text{where}}\,{\text{s}} = \frac{{\text{CI - width}}}{{C{\text{ - alpha}}}}$$

*C* = Number of villages; *P* = 0.54; *D* = design effect = 1.5; *s* = standard error; CI-Width = 0.054; C-alpha = 1.96; *b* = number of households per village = 10.

### Data collection

A team of two medical doctors and two research assistants conducted the data collection. The team visited the randomly selected households and villages. Where a household did not exist because the structure that they had been living in had been demolished, it was excluded from the study. Where study households had moved, the new occupants of the structure were included in the study. Any additional children resident in the selected households were also included, given that they were less than 4 months old or had lived in the household for at least 4 months. Children registered in a household but not known by the respondent were excluded.

A questionnaire-based interview (Appendix 1 in ESM) was carried out with the respondents. Prior to data collection, one medical officer (MO) and two research assistants (RAs) were trained to use a uniform interview technique. The questionnaire was pre-tested before the field work. With the guidance of a community leader, a few homes were sampled, and a questionnaire was administered to parents/ guardians, with their consent. The tool was adjusted based on experience from this field testing to ensure consistency and clarity of the questions.

The information included socioeconomic factors such as respondents’ educational level, literacy and occupation. The number and names of children staying in the household were recorded and compared to the I/M HDSS database list. A standardized medical history for each child residing in the household was then taken (Appendix 1 in ESM). It included questions regarding age, sex, birth circumstances, congenital anomalies, injuries, injury mechanisms, disabilities and treatment. The interviews were conducted in the local language, Lusoga, or English.

Children who were at home underwent a general physical examination by the two doctors (of whom one, MMA, is a general surgeon). Young children were examined in the presence of a parent. Older children were examined without a guardian present as this might compromise their integrity. For children with a reported or detected surgical condition, an additional targeted examination was conducted, aiming to describe further the condition and any associated disabilities (Appendix 1 in ESM). Based on the findings, a recommendation for possible treatment or investigation was made.

At the end of each day, the team reviewed the filled study forms for quality and completeness of data. If there was missing data, issues were discussed with the research assistants and corrected whenever possible.

### Data analysis

The data were entered on Microsoft Excel spreadsheets and analysed using Excel version 16.24. Nominal and ordinal data are presented as counts with percentage. Continuous values are presented with mean and standard deviation for identification of central and variability tendencies. Chi-square test was used to calculate differences between observed and expected outcomes. Student’s *t-*test was used for comparison of means.

### Ethical considerations

The study was granted ethical approval from Makerere University School of Public Health (SNR 542) and approval by the Uganda National Council of Science and Technology (SS 4608). The respondents were interviewed after written or thumb-printed informed consent (Appendix 2 in ESM). Children older than 8 years gave written or thumb-printed assent prior to the physical examination (Appendix 3 in ESM).

All the guardians of children identified with surgical conditions requiring treatment were asked for their willingness for their children to have corrective surgery. If they agreed, information sheets with advice where they could access such surgery were provided. Children in need of more specialized services were referred to an appropriate hospital where these services were offered. While children found with other medical conditions such malaria, skin diseases, sickle-cell disease or worm infestation were sent to the nearest hospital for treatment, and transport money was provided by the PI.

## Results

**Fig. 1:**
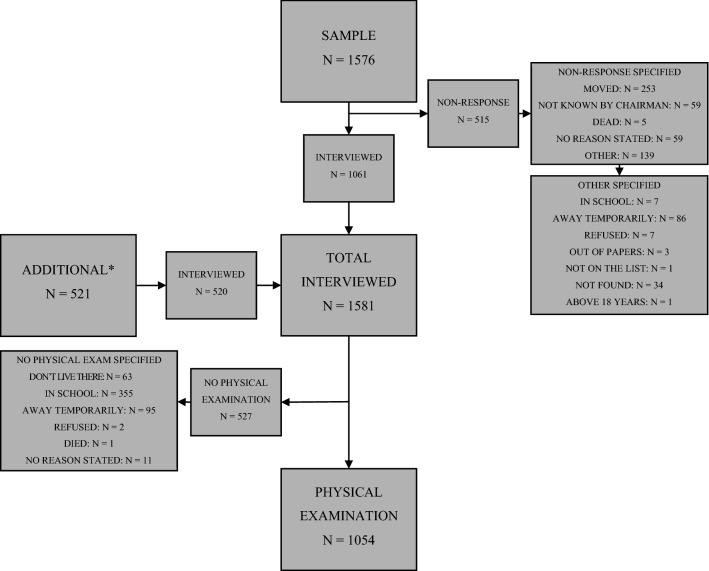
Study participants. *Additional children that had lived in the households for more than 4 months but who were not registered by the I/M HDSS and therefore were not part of the random sample for the study

According to the HDSS registry, some 1576 children resided in the randomly selected 490 households. Of these, data were collected from 357 (72.9%) households. The remaining households could not be found, and some had been demolished. From the households visited, 1061 of the children originally named in the I/M registry-based sample took part in the study. An additional 520 children residing in the study households were also included in the study. The total number of children interviewed was 1581. Of these, 1054 (66.9%) were at home, and were physically examined (Fig. 1).

Table 1 shows the basic characteristics of the households, the children’s guardians and the children included in the study. There were no households without children. The majority (52%) of the households had 4–7 children. Most respondents were literate *n* = 242 (68%), and the most common occupation was peasant (*n* = 214, 60%). The most common place of birth of the children was a Health Centre (*n* = 661, 42%) with a nurse as the birth attendant (*n* = 1296, 82%). Six households (2%) reported one child death each within the last 12 months. The six deceased children were females, and the most common symptom before death was fever (*n* = 5, 83.3%).

**Table 1:** Basic characteristics of the households, the respondents and the children

Households, *n* = 357		Children		
Number of children per household		Age, years	Interviewed, *n* (%) *N* = 1581	Interviewed + examined *n* (%) *N* = 1054
Mean (SD)	4.6 (2.3)	Mean (SD)	8.3 (5.0)	6.9 (4.9)
1–3	128 (36)	<1	77 (4.9)	75 (7.1)
4–7	187 (52)	1–5	466 (29.5)	405 (38.4)
8–14	42 (12)	6–9	354 (22.4)	241 (22.9)
Respondents, *n* = 357	10–14	450 (28.5)	228 (21.6)	
Educational level of respondent, *n* (%)	15–17	234 (14.8)	105 (10.0)	
None	53 (15)	Sex, *n* (%)		
Primary school	193 (54)	Male	812 (51.4)	554 (52.6)
Secondary school	98 (27)	Birthplace, *n* (%)		
Tertiary	10 (3)	At home	216 (13.7)	142 (13.5)
Graduate degree	1 (0)	H/C^a^	661 (41.8)	455 (43.2)
Missing data	2 (1)	Hospital	550 (34.8)	340 (32.3)
Literacy, *n* (%)	Other	154 (9.7)	117 (11.1)	
Literate	242 (68)	Attendant at birth, *n* (%)		
Missing data	2 (1)	TBA^b^	128 (8.1)	80 (7.6)
Occupation of respondent, *n* (%)	Nurse	1296 (82.0)	856 (81.2)	
Unemployed	5 (1)	Doctor	42 (2.7)	38 (3.6)
Student	6 (2)	Other	115 (7.3)	80 (7.6)
Retired	4 (1)			
Home maker	36 (10)			
Peasant	214 (60)			
Self-employed	78 (22)			
Other	12 (3)			
Missing data	2 (1)			
Respondent’s relation to child, *n* (%)				
Mother	747 (45)			
Father	183 (11)			
Grandmother	273 (16)			
Grandfather	60 (4)			
Aunt	107 (6)			
Uncle	37 (2)			
Other	250 (15)			

^a^Health centre

^b^Traditional birth attendant

A total of 186 surgical conditions were detected in 169 children. The prevalence of any surgical condition verified by physical examination was 16.0%, whereas the prevalence of self-reported surgical conditions was 5.4% (Table 2). The prevalence of current unmet surgical need was 6.3% among the children who underwent the physical examination.

**Table 2:** Proportion of children with a verified surgical condition by age and sex

	Untreated		Treated		Total	
	Reported *n* (%) (95% CI)	Verified *n* (%) (95% CI)	Reported *n* (%) (95% CI)	Verified *n* (%) (95% CI)	Reported *n* (%) (95% CI)	Verified *n* (%) (95% CI)
Age in years						
<1	2 (0.1) (0.0–0.3)	14 (1.3) (0.6–2.0)	1 (0.1) (0.0–0.2)	0 (0.0) (0.0–0.0)	3 (0.2) (0.0–0.4)	14 (1.3) (0.6–2.0)
1–5	24 (1.5) (0.9–2.1)	70 (6.6) (5.1–8.1)	8 (0.5 (0.2–0.9)	10 (0.9) (0.4–1.5)	32 (2.0) (1.3–2.7)	80 (7.6) (6.0–9.2)
6–9	13 (0.8) (0.4–1.3)	28 (2.7) (1.7–3.6)	7 (0.4) (0.1–0.8)	9 (0.9) (0.3–1.4)	20 (1.3) (0.7–1.8)	37 (3.5) (2.4–4.6)
10–14	14 (0.9) (0.4–1.3)	18 (1.7) (0.9–2.5)	6 (0.4) (0.1–0.7)	11 (1.0) (0.4–1.7)	20 (1.3) (0.7–1.8)	29 (2.8) (1.8–3.7)
15–17	9 (0.6) (0.2–0.9)	7 (0.7) (0.2–1.2)	1 (0.1) (0.0–0.2)	2 (0.2) (0.0–0.5)	10 (0.6) (0.2–1.0)	9 (0.9) (0.3–1.4)
Sex						
Female	27 (1.7) (1.1–2.3)	59 (5.6) (4.2–7.0)	6 (0.4) (0.1–0.7)	10 (0.9) (0.4–1.5)	33 (2.1) (1.4–2.8)	69 (6.5) (5.1–8.0)
Male	35 (2.2) (1.5–2.9)	78 (7.4) (5.8–9.0)	17 (1.1) (0.6–1.6)	22 (2.1) (1.2–3.0)	52 (3.3) (2.4–4.2)	100 (9.5) (7.7–11.3)
Total	62 (3.9) (3.0–4.9)	137 (13.0) (11.0–15.0)	23 (1.5) (0.9–2.0)	32 (3.0) (2.0–4.1)	85 (5.4) (4.3–6.5)	169 (16.0) (13.8–18.2)

Congenital conditions were the commonest group detected (*n* = 134, 12.7%) followed by trauma-related conditions *n* = 42 (4.0%). Of the congenital conditions, umbilical hernias were the commonest (*n* = 83, 7.8%) followed by groin hernia (*n* = 15, 1.4%) (Table 3).

**Table 3:** Prevalence of surgical conditions among the study participants

	Reported, *n* (%) *N* = 1581	Verified, *n* (%) *N* = 1054
Congenital surgical conditions	54 (3.4)	134 (12.7)
Umbilical hernia	17 (1.1)	83 (7.8)
Groin hernia	6 (0.4)	15 (1.4)
Epigastric hernia		1 (<0.1)
Phimosis	2 (0.1)	0 (0)
Cryptorchidism	4 (0.3)	12 (1.1)
Upper-extremity abnormality	11 (0.7)	11 (1.0)
Lower-extremity abnormality	3 (0.2)	3 (0.3)
Hydrocephalus	3 (0.2)	2 (0.2)
Club foot	1 (<0.1)	1 (<0.1)
Cleft lip	1 (<0.1)	1 (<0.1)
Tetralogy of Fallot	1 (<0.1)	1 (<0.1)
Amniotic band syndrome (amputated fingers and toes)	1 (<0.1)	1 (<0.1)
Hydrocele	1 (<0.1)	1 (<0.1)
Genitalia abnormality	1 (<0.1)	1 (<0.1)
Cystic hygroma	1 (<0.1)	1 (<0.1)
Ankyloglossia	1 (<0.1)	1 (<0.1)
Trauma	19 (1.2)	42 (4.0)
Scars (post-surgery)	17 (1.1)	40 (3.7)
Wound	0 (0)	2 (0.2)
Back injury	2 (0.1)	0 (0)
Other	14 (0.9)	12 (1.1)
Skin condition (skin tag)	3 (0.2)	3 (0.3)
Infectious condition	2 (0.1)	2 (0.2)
Discharging sinus	1 (<0.1)	2 (0.2)
Growth	2 (0.1)	1 (<0.1)
Intraabdominal masses	3 (0.2)	0 (0)
Lipoma	0 (0)	1 (<0.1)
Rectal prolapse	0 (0)	1 (<0.1)
Nasal polyp	0 (0)	1 (<0.1)
Eye conditions (congenital cataract)	1 (<0.1)	0 (0)
Dermoid cysts	0 (0)	1 (<0.1)
Not specified	2 (0.1)	0 (0)

Of the children with a verified surgical condition, 17 (10.1%) had a resulting disability. A disability was reported for 27 children, but it was not clear if this was a result of a surgical or any other type of condition (Table 4).

**Table 4:** Disability in children with surgical conditions

Verified	*N* (%)	Reported	*N* (%)
Minor	7 (4.1)	Disability interferes with normal activities	10 (11.8)
Moderate	7 (4.1)	Disability does not interfere with normal activities	17 (20.0)
Severe	3 (1.8)		
Total	17 (10.1)	27 (31.8)	

Cause of disability in verified cases	*N* (%)
Upper-extremity abnormality	4 (23.5)
Congenital heart condition (surgical)	2 (11.8)
Hydrocephalus	2 (11.8)
Infectious condition	2 (11.8)
Cleft lip	1 (5.9)
Clubfoot	1 (5.9)
Amniotic band syndrome	1 (5.9)
Back injury	1 (5.9)
Birth injury	1 (5.9)
Burns	1 (5.9)
Wound	1 (5.9)

## Discussion

In this study, the prevalence of surgical conditions in children was high at 16.0% and 6.3% had a current unmet surgical need at the time of the study. Some 10.1% had some degree of disability verified through physical examination. This indicates a significant amount of morbidity and suffering in the cohort.

Previous research on paediatric surgical conditions has relied mostly on self-reported surgical need through questionnaire-based surveys. One of these studies was a nationwide household survey, conducted in Uganda in 2016 [3]. That study showed a prevalence of 14% of surgical conditions and half of the children had received surgical treatment. The unmet paediatric surgical need was estimated at 7.2%. Epidemiological research conducted in Rwanda, Sierra Leone, Nepal and Uganda has shown an overall surgical need of 19%. This figure is similar to that in the present study where the prevalence of surgical conditions in children was 16%. The discrepancy between self-reported conditions and those verified through physical examination can be a result of lack of awareness in the community as well as among health care professionals.

The prevalence numbers in this study extrapolated to the general population in Uganda below 15 years of age (19.7 million) would result in 3.1 million children with a surgical condition at some point during their childhood. The current need for surgery is estimated to 1.2 million. The youngest children had the highest prevalence of surgical conditions in our cohort. This finding is important from the health care systems perspective as children in these age groups are susceptible to several non-surgical conditions as well. To ensure good health for them, safe delivery of surgical services would be a prerequisite.

Congenital malformations represented the largest group of conditions identified in the present study. Congenital anomalies are the fifth leading cause of death in children under 5 years of age and those that are not immediately lethal contribute to long-term disability with subsequent effects on the individual, family and economy [11–13].

Morbidity due to congenital anomalies, trauma and other acquired conditions in children in LMICs is, however, largely unknown [3]. A previous study estimated that 7.1% of Uganda’s population had a disability and that 31% of them were children, but that study included all types of disability [14]. The present study documented disability in 10.1% of the children with a surgical condition.

Long travel distances, lack of money and fear of surgery represent important barriers to accessing surgical care [15]. Others include reluctance among healthcare providers to treat infants, and lack of trained specialists in paediatric surgery to manage children’s surgical conditions [16]. A recent qualitative study on barriers to surgical care for children in Uganda found that hernia in children is often overlooked and that these children are treated for other conditions instead. Collaboration between health care professionals and community leaders to increase awareness plus the institution of teams dedicated to paediatric surgery were suggested ways forward [16].

Given the monumental burden of disease due to surgical conditions in children, paediatric surgical services must be prioritized in the work towards universal health coverage. There must be a rapid upscaling in surgical facilities and staff in the children’s vicinity, to meet the current and the future surgical need. Otherwise, the SDG 3 will not be achieved.

### Strengths and limitations

A limitation of this study was that about one third of the original sample was not found. The most common reason for non-response was that the children had moved out from the I/M HDSS. This was compensated for statistically through a design effect of 1.5. In addition, many children residing in the households not registered by the I/M HDSS were detected and included in the study. This was a result of high turn-over of I/M HDSS residents. There is no reason to believe that moving from the I/M HDSS or being born into a I/M HDSS household but not being registered is related to any paediatric surgical condition.

The internal validity of the results is high and should be accurate for the I/M HDSS as a whole. The households and thereby the children to participate were random-sampled, to avoid selection bias. In addition, the study sample was relatively large and represented both urban (town) and rural locations. The physical examination by medical doctors, of whom one was a surgeon, makes the results more reliable than if only interviews had been carried out.

This study aimed to define the prevalence of surgical condition and associated surgical need in the paediatric population in the study area. Further knowledge about the incidence of surgical conditions, in particular emergent surgical conditions, will be necessary in order to plan for healthcare services that can cater for the overall surgical need in the paediatric population.

## Conclusion

This study has shown that surgical conditions are common in children in eastern Uganda. This represents a significant amount of suffering over time and will often cause disability and premature death. With a growing population, the country’s need for paediatric surgical capacity will increase even further. The health care system must be reinforced to also be able to provide services for children with surgical conditions if Sustainability Development Goal 3 is to be achieved.

## Supplementary Information

Below is the link to the electronic supplementary material.

Supplementary file1 (DOCX 18 KB)

Supplementary file2 (DOCX 25 KB)

Supplementary file3 (DOCX 156 KB)
