# Supplementary material for: Prevalence of Paediatric Surgical Conditions in Eastern Uganda: A Cross-Sectional Study
Source: World J Surg. 2022 Jan 1;46(3):701–8. doi: 10.1007/s00268-021-06378-9 (PMC8803710; doi:10.1007/s00268-021-06378-9)
Supplement: Supplementary file 1 — Supplementary file1 (DOCX 18 KB) [file 268_2021_6378_MOESM1_ESM.docx]

A. Identification of the informant (parent/guardian)

1. Name of district ***_________________________________________***
   1. IGANGA
   2. MAYUGE
   3. BUGWERI

1. Parish Name__________________________

1. Village name***______________________***

1. Household code: ___________________________________

(01, 02, 03………………………10)

1. Local Chairman’s name & telephone contact

____________________ _____________________

1. Name of respondent

______________________________________________________________________

B. Information about the respondent

1. ***Obwegerese obwoyo gw’olikubuuza ebibuzo bino***

***: Wasoma wakoma mu kibbina ki, oba oli ni kooosi yolikusoma buti ?***

*What is the highest educational level that you have achieved or are currently following?*

1=None (includes nursery), 2=Primary school, 3=Secondary school (junior / senior), 4=Tertiary (diploma, colleges, bachelors), 5=Graduate degree (Master degree, PhD)

1. ***Okusoma n’okughandiika :***

***Waliwo olulimi Lw’oidhi okusoma n’okuluwandiika ? ( Abantu abakulu n’abaana abato abalikwega obwege okughandiika , bairamu bati bbe )* Literacy:**

*Are you able to read and write in any language*? Y/N

*[For adults and children who are currently learning how to read and write answer: ‘No’]* Yes/ No

1. ***Omulimu Gwakola :***

***Mulimu ki gwokola buli lunaku okwebesawo ?***

**Occupation:**

*What is your primary occupation?*

1=Unemployed (looking for a job), 2=student, 3=retired, 4= home maker [Housewives],

5=peasant, 6=self employed, 7=other (specify)________________________________

1. ***Abaana abakali kuweza myaaka 18 egyobukulu abali mu maka gano :***

***Bita mu lisiti eya Abaana abali mu buli nhumba wetegereze obone oba nga ntuufu.***

***Waliwo abaana abeyongeireku okuswiika ku lisiti yebatuwa ku lule?.Oba bwekiri wandiika amaina gaibwe wano :***

**Number of children under the age of 18 in the household:**

*Make a list of all children in the household below.*

| Study ID | HDSS/dummy number | Respondents relation to child * | Name of child | Age (M/Y) | Sex (F/M) |
| --- | --- | --- | --- | --- | --- |
|  |  |  |  |  |  |
|  |  |  |  |  |  |
|  |  |  |  |  |  |
|  |  |  |  |  |  |
|  |  |  |  |  |  |
|  |  |  |  |  |  |
|  |  |  |  |  |  |
|  |  |  |  |  |  |
|  |  |  |  |  |  |
|  |  |  |  |  |  |
|  |  |  |  |  |  |
|  |  |  |  |  |  |
|  |  |  |  |  |  |
|  |  |  |  |  |  |
|  |  |  |  |  |  |
|  |  |  |  |  |  |
|  |  |  |  |  |  |
|  |  |  |  |  |  |
|  |  |  |  |  |  |
|  |  |  |  |  |  |

****Buzaleki / Lugandaki Ighe / Imwe lwemulinalwo N’abaana abali mu maka gano ?***

*Respondents relation to the children in the household:*

1=mother, 2=father, 3=grandmother, 4=grandfather, 5=aunt, 6=uncle, 7=other (specify)

1. ***Waliwoku Abaana abafiire mu maka gano okuva mu myezi eikumi nebiri egibise?***

***11a.Bwekiba nga kituufu, Wandiika amaina gaibwe ghano :***

**Have any children in this household died in the last twelwe months?** *11a. If yes, fill the table below:*

| **Name** | **Month of death** | **Age at death** | **Sex** | **Cause** |
| --- | --- | --- | --- | --- |
|  |  |  |  |  |
|  |  |  |  |  |
|  |  |  |  |  |
|  |  |  |  |  |
|  |  |  |  |  |
|  |  |  |  |  |

***11b. Omwaana ono nga akali kufa , mu kiseera ekitaswika wiiki, yalaga kabonero ki kubono?***

**11b. Did the child have any of the following problems less than a week before she/he died?**

1=Injury, 2=Wound not due to an injury, 3=Bleeding or ill around childbirth, 4=Mass (Growth or Swelling), 5=Deformity congenital, 6=Abdominal distention or pain, 7=other (specify

___________________________________

***11c*. *Abaana abawere N’abato einho ;***

***:Omwaana yali aboneka atya nga yakazalibwa : yali nga asobola okunhwa , era nokufuka nokupama bukalamu?* Y/N**

**For neonates / babies: Did the child look normal and could it drink, urinate and defecate normally after birth? Y/N**

***11d.*** ***Nkusaba onkobereku byoidhukira ku kufa kwo omwana ono***

*Briefly tell me what you can remember about the death of the child*

___________________________________________________________________________

___________________________________________________________________________

___________________________________________________________________________ ___________________________________________________________________________

___________________________________________________________________________

C. Medical history (fill one per child)

***Eby’obulamu ebigema ku buli Mwaana ali mu maka gano :***

***Erina Er’yomwaana***

Medical history for each child in the household

Code of child________________________________________

| 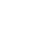 | 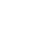 |
| --- | --- |

***12a.*** Age years ***12b*.** Sex F/M

1. ***Omwana bamuzalira wa?***

*Where was the birth of the child?*

1= at home, 2=H/C, 3=Hospital, 4= other (Specify) _________________________

1. ***Ani eyakuzalisa?***

*Who attended to you?*

1= traditional birth attendant, 2.=Nurse, 3=Doctor, 4=Other

specify___________________________________________________________________

1. ***Omwana bamubonaku obulemu?***

*Was any abnormality of the child detected between the delivery and when you returned back home? (Y/N)*

1. ***Bakukobera nti bamubweineku obulema?***

*Were you told that the child had an abnormality? (Y/N)*

***16a. Oba nga kityo bwekyaali, obulema bwali wa ku mubiri?***

*If yes, which body part was affected?*

1=head/face, 2=back, 3=abdomen, 4=arms and hands, 5=legs and feet, 6=groin, genitalia and anus

***16b.Bwewabayo embeera eyindi, Ginhongole***

Specify the condition ________________________________________________________________________

__________________________________________________________________________________________

__________________________________________________________________________________________

***16c. Bwewaba nga yali n’obuzibu ,Yafuna obwidhandhabi ?***

*If yes, did ……………… receive any treatment for the condition? Y/N*

***16d. Yafuna bwidhandhabi bwa ngeri ki ?***

*If yes, which type of treatment*

1=surgery, 2=medicines, 3=herbs, 4=other (Specify)…………………………..

***16e. Bwabanga bee lwaki omwana tiyafuna bwidhandabi?***

*If no, why didn’t the child get any treatment?*

________________________________________________________________________

__________________________________________________________________________________________

__________________________________________________________________________________________

***16f*. *Wandienze omwana alongosebwe, aterere?***

*Are you willing for your child have a corrective surgery, if required? Y/N*

***17a. Omwana ono yafunakuku obukosefu?***

*Has this child ever had an injury? Y/N*

***17b. Obukosefu obwo bumuletera obuzibu?***

*Does the injury still cuasing him/her any problem? Y/N*

**17c. *Oba nga niikyo, bukosefu ki?***

*If yes, which kind of injuries?*

1=Fracture of upper extremity, 2=fracture of lower extremity, 3=burn, 4=other type of injury

(specify)___________________________________________________________________________________

__________________________________________________________________________________________

**17d. *Oba nga kituufu, ya funa atya obukosefu buno***

*If yes, how did the injury occur?*

1=fall, 2=road traffic accident, 3=hot water/oil/fluids, 4=fire, 5=other (specify)_______________________

_______________________________________________________________________________________

**17e. *Oba nga kituufu yafuna bwindhandhabi bwona bwona??***

*If yes, did ……………… receive any treatment for the condition? Y/N*

**17f. *Oba nga yyi, bwidhandhabi ki?***

*If yes, which type of treatment*

1=surgery, 2=medicines, 3=herbs,

4=other (Specify)____________________________________________________________________

**17g. *Oba nga bbe, lwaki tiyafuna bwidhandhabi?***

*If no, can you tell us why the child did not receive treatment?*

___________________________________________________________________________

___________________________________________________________________________

_________________________________________________________________________

***18a. (Name) .... alina obulema bwona bwona?***

| Does ……... have any disabilities? |  |
| --- | --- |
|  |  |
| 1. Disability with a need for help with all activities (moving around, eating, hygiene, going to the toilet etc) | □ |
| 2. Disability that necessitates immobility (sitting/Lying, cannot move) | □ |
| 3. Disability that interferes with most activities (play, school, physical activity/mobility) | □ |
| 4. Disability that cannot be ignored and that interferes with concentration on school work, chores and daily activities | □ |
| 5. Disability that cannot be ignored but that does not interfere with everyday activities (playing, going to school or chores at home | □ |
| 6. Minor disability that can be ignored | □ |
| 7. No disability | □ |

***18b. Obutesobola bumuletera okuswaala, ekimulemesa okwe nyhigira mu mirimu nibaine abe myaka gye?***

*If yes, does the disability cause embarassment/shame which limits the possibilities for …….. to participate in normal activities for her/his age ?* Y/N

***19a. …. Bamulongosaaku?***

*Has ………. ever been operated?* Y/N

**19b. *Oba nga yyi, bamulongosaaki?***

*If yes, for what condition(s)*

1=groin hernia, 2=circumcision, 3=abdominal condition, 4=abscess, 5=other (specify)

_________________________________________________________________________

**19c. *Oba nga kituufu, …. Yali n’emyaka/emyeezi emeka?***

*If yes to question 19a, how old was …….. at the time of the surgery?*

___________________________________________________________________________

**19d. *Oba nga kituufu ba mulongosaaku, bamulongosezawa***

*If yes at question 19a, where was the procedure performed*

*name of facillity________________________________________________*

1=Hospital, 2=Other public health facility, 3=Mission hospital, 4=Private clinic, 5= Do not know, 6=Other location (specify) ..........................

**19e. *Oba nga bbe, lwaaki tibamulongosa?***

*If no, can you tell us why the child did not get treatment?*_________________________

_________________________________________________________________________

_________________________________________________________________________

_________________________________________________________________________

_________________________________________________________________________

_________________________________________________________________________ _________________________________________________________________________

**20. *Bwewabawo ebindi eby’kwongeraku***

*Other comments*

___________________________________________________________________________

___________________________________________________________________________

___________________________________________________________________________

___________________________________________________________________________

___________________________________________________________________________

D. Physical Examination

21. Code of child________________________________________

| **Examination performed by**    **_______________________** |
| --- |

**22. Date of examination**  **(dd/mm/yy)**


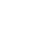

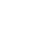

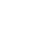

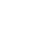

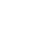

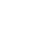


General physical examination

| 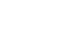 | 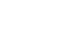 |
| --- | --- |

| 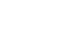 | 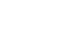 | 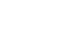 |
| --- | --- | --- |

23. Weight in kg

24. Height in cm

| 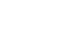 | 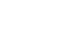 |
| --- | --- |

25. Head circumference in cm

26. Head (check for any swellings/masses, deformity, scars and inside the mouth) 1=normal, 2= abnormal finding (specify)________________________________

1. **Neck (check for any swellings/masses, deformity)**.

1=normal, 2= abnormal finding (specify)________________________________

1. **Back (check for abnormalities, sweelings/masses, scars)**

1=normal, 2= abnormal finding (specify)________________________________

1. **Chest- (check for deformity, swellings/masses, scars)**

1=normal, 2= abnormal finding (specify)_______________________________

1. **Respiratory system – Difficulty in breathing, check for normal air entry**

1=normal, 2= abnormal finding (specify)_________________________________

31. Abdomen (check for deformity, swelling/masses, scars)

1=normal, 2= abnormal finding (specify)_________________________________

32. Groin, check for the following conditions

1= normal finding, 2=scar after previous hernia surgery, 3=groin hernia unilateral, 4=groin hernia bilateral, 5= other (specify)_____________________________________

33. Genitalia

1=normal finding, 2=scrotal/labial hernia, 3=undescended testis unilateral (Male child), 4= undescended testis bilateral( Male child) 5= other

(specify)_______________________________________

**34. Extremities (check for deformity, mal-healed fractures, chronic osteomyelitis, swellings/tumours)**

1=normal, 2= abnormal finding (specify)_________________________________

35. Comments / Recommendation (any treatment recommended, how and by whom it will be done)

___________________________________________________________________________

___________________________________________________________________________

___________________________________________________________________________

___________________________________________________________________________

___________________________________________________________________________

___________________________________________________________________________

___________________________________________________________________________

___________________________________________________________________________

___________________________________________________________________________

___________________________________________________________________________ ___________________________________________________________________________

E. Targeted physical examination – base on results in interview and general physical examination

36. The examination refers to question ______________

1. **Describe and name the condition:**

___________________________________________________________________________

___________________________________________________________________________

___________________________________________________________________________

___________________________________________________________________________

___________________________________________________________________________ ___________________________________________________________________________

1. **Does the condition cause any disability? Y/N**

39a. If yes, what kind(s) of disability

1=problem walking, 2=problem standing, 3=problem sitting, 4=problem with communication, 5=problem eating, 6=problem with fine motorics/handling pencil and similar, 7=problem extending/contracting arms and legs, 8=Other___________________________________

39b. If yes, fill the table below

| Type of disability | Extent of disability  1=minor, 2=moderate,  3=severe, |
| --- | --- |
|  |  |
|  |  |
|  |  |
|  |  |
|  |  |

39c. Describe in free text the disabilities that ____________ has (type of disability and extent of disability)

___________________________________________________________________________

___________________________________________________________________________

___________________________________________________________________________

___________________________________________________________________________

___________________________________________________________________________

___________________________________________________________________________

___________________________________________________________________________ ___________________________________________________________________________

40: Recommendation (any treatment recommended, how and by whom it will/can be done)

___________________________________________________________________________

___________________________________________________________________________

___________________________________________________________________________

___________________________________________________________________________

___________________________________________________________________________

___________________________________________________________________________

___________________________________________________________________________

___________________________________________________________________________

1. **Examination done by ___________________________________________**
2. **Photo taken (Y/N))**

Photo file number and code__________________________________________
