# Supplementary material for: Prevalence of Paediatric Surgical Conditions in Eastern Uganda: A Cross-Sectional Study
Source: World J Surg. 2022 Jan 1;46(3):701–8. doi: 10.1007/s00268-021-06378-9 (PMC8803710; doi:10.1007/s00268-021-06378-9)
Supplement: Supplementary file 2 — Supplementary file2 (DOCX 25 KB) [file 268_2021_6378_MOESM2_ESM.docx]

**Appendix 2.**

**Consent form for the paediatric surgery prevalence study**

**Introduction:** My name is ………………………., and I am part of the team conducting a study about surgical conditions in children.

**Purpose:** Children may be born with or may develop conditions that require surgical intervention for their treatment. Examples of such conditions are groin hernia and hydrocele, fractures and burns. Groin hernia is among the most common surgical conditions seen both in children and in adults. Among adult men in the I/M HDSS almost 10% had or had been treated for a groin hernia. The purpose of this study is to investigate how common surgical conditions are among children in the I/M HDSS. We particularly focus on groin hernia and disability due to previous fractures and burns but other conditions, if existing, will also be recorded.

**Research procedure:** We will interview and examine 900 children together with their guardians. This will require 10- 20 minutes per child. We will sometimes require to take photos to describe the characteristics of the surgical conditions or for illustration of the surgical problem that is unique. For such cases we will take a photo, but measures will be put in place so as not to expose the patient’s identity.

**Potential benefits from this study:** For patients who are found to have a surgically correctable anomaly or sequela after injuries, we will need to talk to the parents about health seeking behaviour. We will ask for their willingness for their children to have a corrective surgery. If they agree information sheets with advice where they can get treatment using the most accessible area in the district will be provided.

**Potential harm from this study:** There are no risks associated with your participation in this study. There are no interventions involved. For those who are in need of a surgery, there are always risks related to surgery. These risks are not elevated compared to undergoing surgery elsewhere or by someone else.

**Confidentiality and autonomy:** the information that you give us will be kept safely so that no one apart from the investigators have access to it. All data will be de-identified so that your name will not appear in the analysis or any resulting publications. If you have any questions about the study, please ask me. Also Dr. Ajiko Mary Margaret whose phone number is +256-772413810 can be contacted for further questions. It is your decision to participate in the study and you are free to stop, without any consequences, at any time.

Questions regarding the rights of participants or any complaints about the research can be directed to Dr. Suzanne Kiwanuka, the chair person of the School of Public Health Research and Ethics Committee, telephone number 0701-888-163 or 0312-291-397, e-mail address [skiwanuka@musph.ac.ug](mailto:skiwanuka@musph.ac.ug).

**Acceptance:** Before we proceed with the interview, I would like to seek your permission. Do you consent to participate in the study?

**Voluntary participation:** Participation in this study is voluntary. If you choose to take part, I will first ask you to sign or finger print this document to verify your consent to participate, and can keep a copy of the consent form.

I have been fully explained about this study and understand its purpose and objectives. I understand the details and have been informed about the requirements of the study. My questions have been answered satisfactory. I hereby agree to participate in the study.

Signature of respondent ___________________________________ Date________________

Thumb print of respondent ----------------------------------------------

Signature of interviewer___________________________________ Date________________
