# Supplementary material for: Prevalence of Paediatric Surgical Conditions in Eastern Uganda: A Cross-Sectional Study
Source: World J Surg. 2022 Jan 1;46(3):701–8. doi: 10.1007/s00268-021-06378-9 (PMC8803710; doi:10.1007/s00268-021-06378-9)
Supplement: Supplementary file 3 — Supplementary file3 (DOCX 156 KB) [file 268_2021_6378_MOESM3_ESM.docx]

**Appendix 3.**

**Assent form paediatric surgery prevalence study**

**Introduction:** My name is ………………………., and I am part of the team conducting a study about surgical conditions in children.

**Purpose:** We are doing a research study about diseases in children that are treated by operation. A research study is a way to learn more about people. If you decide that you want to be part of this study you will be asked a few questions about your health. In this study we will ask you if you have any swellings in your body, some of the swellings are born with, others you get them when you get injuries such as falling from trees, or burns, or during sports at school. We are interested in those children with hernia and how your parents did in order to bring you to this hospital.

**Research procedure:** There are some things about this study you should know. In these procedures we will need to examine you to find out if you have hernia. This will require 10- 20 minutes and may cause you discomfort. We will sometimes require to take photos if we find that you have a condition that is not common, but we will not expose your face so that people will not recognize you. These photos will be used for discussion with other doctors how to treat your disease well. If you accept us to take your photo you will be required to sign a special photo consent form.

**Potential benefits from this study:** Not everyone who takes a part in this study will benefit. A benefit means that something good happens to you. We think these benefits might be description.

**Potential harm from this study:** There are no risks associated with your participation in this study. No injections, and we will not inflict pain to you, but we will only ask questions and examine you.

**Confidentiality and autonomy:** When we are finished with this study we will write a report about what we learnt. This report will not include your name or that you were in the study.

**Acceptance:** You do not have to be in the study if you do not want to be. If you decide to stop after we begin, that is okay too. Your parents know about the study too.

Also Dr. Ajiko Mary Margaret whose phone number is +256-772413810 can be contacted for further questions. Questions regarding your rights to take part of this study or any complaints about the research can be directed to Dr. Suzanne Kiwanuka, the chairperson of the School of Public Health Research and Ethics Committee, telephone number 0701-888-163 or 0312-291-397, e-mail address [skiwanuka@musph.ac.ug](mailto:skiwanuka@musph.ac.ug).

If you decide to be in the study, please sign your name.

Your signature__________________________________ Date________________

Your thumb print ----------------------------------------------

Signature of interviewer___________________________________ Date________________
